# Editorial: Immunotherapy for NSCLC with oncogenic driver variants

**DOI:** 10.3389/fonc.2022.1095947

**Published:** 2022-12-08

**Authors:** Yijia Du, Qian Chu, Yanyan Lou, Yong He, Hongbo Hu, Qipeng Hu, Meijuan Huang

**Affiliations:** ^1^ Thoracic Oncology Ward, Cancer Center, West China Hospital of Sichuan University, Chengdu, Sichuan, China; ^2^ Department of Oncology, Tongji Hospital, Tongji Medical College, Huazhong University of Science and Technology, Wuhan, China; ^3^ Division of Hematology and Medical Oncology, Mayo Clinic, Jacksonville, FL, United States; ^4^ Department of Respiratory Medicine, Daping Hospital, Army Medical University, Chongqing, China; ^5^ Department of Rheumatology and Immunology, State Key Laboratory of Biotherapy and Collaborative Innovation Center for Biotherapy, West China Hospital, Sichuan University, Chengdu, China; ^6^ Division of Pathogenesis of Virus Associated Tumors, German Cancer Research Centre Deutsches Krebsforschungszentrum(DKFZ), Heidelberg University, Heidelberg, Germany

**Keywords:** oncogenic driver variants, tumor immune microenvironment, lung cancer, targeted therapy resistance, immune checkpoint inhibitors

Immune checkpoint inhibitor (ICI) has been recognized as a gold standard treatment for advanced non-small cancer (NSCLC) without driver variants (1). However, the activity of ICIs across NSCLC harboring oncogenic alterations (such as EGFR, ALK, ROS1, or BRAF) is poorly characterized (2). Herein, we set this Research Topic to broadly collect research regarding immunotherapy for advanced NSCLC with oncogenic mutations, expecting to explore the role of immunotherapy (such as ICIs or in combination with other therapies) in these patient populations.

We would like to thank all authors for their contribution to providing further evidence regarding the treatment timing and treatment option with ICIs in the mutation population. Considering the high toxicity and low therapy efficacy of administrating ICIs as the first-line treatment of driver gene-positive NSCLC, the research reported on the Topic mostly focused on the application of ICIs in the ≥ second-line treatment setting (3, 4). What is more, Tian et al. reported that patients who received subsequent ICIs after progression on tyrosine kinase inhibitors (TKIs) achieved a higher quality of survival benefits compared with those who received ICI as later lines treatment. Moreover, Zhai et al. reported the re-sensitization to TKIs after pembrolizumab resistance in a non-smoker patient carrying EGFR 19 deletions (19del) who received previous targeted therapy. In terms of treatment options, the investigators also revealed the encouraging efficacy of ICI-based combination therapy especially in combination with chemotherapy, which was consistent with other studies. The platinum-based chemo-immunotherapy combination provides a greater survival advantage compared with single-agent therapy for patients with EGFR mutation (Tian et al.). In addition to the classical platinum-based chemotherapy, platinum-free chemotherapy combined with ICI is associated with favorable progression-free survival in patients with EGFR-TKI-resistant advanced NSCLC from the retrospective study by Deng et al.

The authors move on to discuss the potential beneficiaries of ICIs therapy and found that the genetic and immunological diversity of the various mutational subtypes may possess different prognostic. Lei et al. indicated that several uncommon EGFR mutation subtypes (such as S768I, T790M, G718A, LL861Q, G719C, and 20ins) had a higher proportion of tumor mutational burden (TMB)-high or strong positive programmed death-ligand 1 (PD-L1) expression than the total EGFR mutation group (Leu858Arg [L858R] and 19del) by analysis of 9649 Chinese patients with primary NSCLC. Besides, a case report from Peng et al. demonstrated that ICIs may be more effective for *EGFR* L858R mutation than for other *EGFR* mutant subtypes, which is related to some potential predictors, such as TMB and concurrent PD-L1 plus CD8^+^tumour-infiltrating lymphocyte (TIL) expression. Meanwhile, patients with *EGFR* mutations and higher PD-L1 expression are more likely to obtain potential benefits from immunotherapy after TKIs resistance (Zhai et al.). However, an EGFR mutant NSCLC case with high PD-L1 expression showed resistance to chemo-immunotherapy in an “immune-cold” microenvironment (Zhao et al.). Moreover, Dong et al. showed that an *ALK*-positive NSCLC patient with multiple driving mutations (*BRAF, KRAS, PIK3CA*), high TMB, PD-L1 overexpression, and CD8^+^TIL may benefit from nivolumab.

To our knowledge, there are extremely limited data on the use of immunotherapy in populations of other gene aberation compared to EGFR. Therefore, collecting more evidence is necessary to confirm the efficacy of immunotherapy in these populations. The authors published in this “Research Topic” presented some populations containing oncogenic driver variants that benefit and lack of benefit from ICIs or high risk of toxicities or hyper-progression of anti-PD-1/anti-PD-L1 therapy. Zhou et al. performed whole exome sequencing in a cohort of 33 Chinese patients with NSCLC and identified that NSCLC tumors harboring mutated mucin 19 mutation exhibited good responses to anti-PD-1 inhibitors. Besides, Yang et al. demonstrate that endoplasmic reticulum aminopeptidase 2 (ERAP2) was lowly expressed in squamous cell lung carcinoma (SqCLC) and was significantly associated with longer survival. In addition, patients with higher oxidized low density lipoprotein receptor 1(OLR1) expression were predicted to have better immunotherapy outcomes based on Gene Expression Omnibus (GEO) data mining in the study by Liu et al. Moreover, a 5-genomic mutation signature could predict the survival of patients with NSCLC receiving atezolizumab (Lin et al.) and co-occurring alteration of NOTCH and DDR pathways served as a novel predictor to efficacious immunotherapy in NSCLC (Zhang et al.). In contrast to the above-mentioned studies, another study found that high expression of nsulin-like growth factor-I (IGF-1) was closely bound up with the unfavorable overall survival for patients with bladder urothelial carcinoma (BLCA), cholangiocarcinoma (CHOL), and acute myeloid leukemia (LAML) based on Cox regression analysis and Kaplan-Meier survival analysis (Zhang et al.). Besides, Zheng et al. found secretory phosphoprotein 1 (SPP1) expression was higher in patients with EGFR mutation and its high expression was associated with poor prognosis.

In conclusion, setting the “Research Topic” of “*Immunotherapy for NSCLC with Oncogenic Driver Variants*” to publish relevant research is an extraordinary and timely effort. We have tried to uncover more treatment details on the application of immunotherapy for EGFR-mutant, as well as to report more novel efficacy-related gene aberration that benefit from ICIs or lack of benefit of anti-PD-1/anti-PD-L1 therapy. We truly believe that these efforts will be beneficial for us to build a clearer picture of the role of ICIs for NSCLC with oncogenic driver variants and greatly enhance existing treatment strategies to maximize patient benefit.

## Author contributions

All authors listed have made a substantial, direct, and intellectual contribution to the work and approved it for publication.
